# Sensory Evaluation of a Receptor-Guided Mixture Designed to Approximate Methyl Eugenol Odor Quality

**DOI:** 10.3390/foods15213963

**Published:** 2026-11-04

**Authors:** Shoji Ogami, Yuhi Hattori, Yosuke Fukutani

**Affiliations:** 1 San-Ei Gen F.F.I., Inc., 1-1-11 Sanwa-cho, Toyonaka 561-8588, Japan; shoji-ogami@saneigenffi.co.jp (S.O.); yuhi-hattori@saneigenffi.co.jp (Y.H.); 2 Department of Biotechnology and Life Science, Tokyo University of Agriculture and Technology, Koganei 184-8588, Japan

**Keywords:** flavor formulation, olfactory receptor, Napping, check-all-that-apply, perceptual similarity, dynamic headspace

## Abstract

Reconstructing the odor quality of a target compound using alternative aroma ingredients remains a challenge in flavor science and is largely guided by empirical sensory evaluation. As a proof of concept, this study investigated whether human olfactory receptor response patterns can inform candidate selection using methyl eugenol as a model odorant. Functional assays confirmed that methyl eugenol activated OR10G7, OR5K1, and OR2J2, whereas its structural analog, eugenol, predominantly activated OR10G7. To address receptor-response differences between methyl eugenol and eugenol, 2,5-dimethylpyrazine and (*E*,*E*)-2,4-decadienal were selected through preliminary sensory evaluation and combined with eugenol to formulate a mixture designed to approximate the target odor quality (Mix). In preliminary exploratory assessments involving six panelists over two sessions, Mix was selected as more similar to methyl eugenol than eugenol in 11 of 12 panelist-session observations in the 2-AFC test; the distance evaluation showed the same directional pattern. Napping and check-all-that-apply (CATA) profiling were descriptively consistent with a directional shift of Mix toward methyl eugenol; notes were not fully reproduced. Dynamic headspace GC–MS analysis showed that eugenol predominated in the DHS-recovered fraction. Collectively, these findings suggest that olfactory receptor response patterns can provide a mechanistically informed basis for narrowing candidate selection, while sensory evaluation can be used to assess whether the resulting mixture perceptually approximates the target odor quality.

## 1. Introduction

The reconstruction of a specific odor generally relies heavily on the experience of flavorists and an iterative sensory evaluation. Although this empirical approach is practically effective, the number of available flavor ingredients and their possible combinations is enormous, making it difficult to efficiently identify an optimal formulation. Furthermore, sensory optimization alone provides limited mechanistic insights into why a particular mixture resembles the target odor. Therefore, design principles that enable a more rational selection of candidate compounds would be valuable for odor reconstruction.

To examine such a design strategy in a practically relevant and experimentally tractable system, we selected methyl eugenol (MetEug) as a model odorant. Methyl eugenol is an important odorant with spicy, clove-like, and phenolic characteristics that contribute to the characteristic aromas of various flavor and fragrance materials, serving as a major phenylpropanoid constituent of natural essential oils derived from sweet basil and tarragon [1,2]. However, toxicological concerns have been raised about methyl eugenol. It is listed as a substance reasonably anticipated to be a human carcinogen in the 15th edition of the U.S. National Toxicology Program Report on Carcinogens [3] and as probably carcinogenic to humans (Group 2A) in volume 134 of the IARC Monographs [4]. In addition, the direct addition of synthetic methyl eugenol to food is subject to regulatory restrictions in Europe and the United States [5]. Therefore, reproducing the odor quality of methyl eugenol using mixtures of alternative compounds represents both a commercially critical replacement challenge and an ideal model system, particularly because receptor-response information for methyl eugenol and its structural analog eugenol is already available.

The response profiles of human olfactory receptors (ORs) may provide a promising framework for the design principle. The relationship between ORs and odorants is many-to-many; an individual OR can respond to multiple odorants, whereas a single odorant can activate multiple ORs [6]. Accordingly, odor quality is thought to be represented, at least in part, by combinatorial patterns of activity across the OR repertoire [7]. Humans possess approximately 400 potentially functional OR genes, whose overlapping ligand-response profiles enable the discrimination of a vast array of environmental odorants [8,9]. However, receptor responses to odorant mixtures are not necessarily a simple summation of the responses to individual components. Antagonism, partial agonism, and other forms of receptor-level interactions can substantially alter the ensemble response evoked by a mixture [7,10,11,12]. Importantly, such receptor-level interactions have been shown to alter human odor perception, demonstrating that OR response patterns can directly contribute to the perception of odor mixtures and provide useful insights into individual and qualitative differences in odor perception [13].

Functional variations in the human OR repertoire have also been associated with individual differences in odor sensitivity and perceived odor quality. Moreover, an increasing number of human OR-responsive food- and flavor-related odorants have been identified, and OR assays have been utilized to investigate odor masking and the interactions among odorants [12,13,14,15]. Together, these findings support the use of OR response patterns as molecular indicators for designing complex odor blends. Nevertheless, few studies have directly used differences in receptor activity as the starting point for selecting components of substitute flavor mixtures and subsequently validated the resulting mixtures through both sensory analysis and headspace characteristics.

Therefore, we hypothesized that for structurally related odorants, receptor activities elicited by a target odorant but absent or weaker in a congener could be complemented by auxiliary compounds that activate the corresponding receptors. Eugenol (Eug) is structurally similar to methyl eugenol and shares its primary spicy and clove-like characteristics [16]; however, the two compounds remain perceptually distinct. Methyl eugenol has also been described using hay-like, dried-grass, and woody descriptors [16,17,18]. The differences between the two compounds are also reflected at the receptor level: methyl eugenol activates OR10G7, OR5K1, and OR2J2, whereas eugenol activates OR10G7 but exhibits only weak activity toward OR2J2 and no confirmed activity toward OR5K1 [9,19,20]. These differences suggest that eugenol could serve as a structural and sensory reference for methyl eugenol reconstruction, while the missing OR5K1- and OR2J2-associated response components could be supplied by auxiliary odorants.

**Figure 1:**
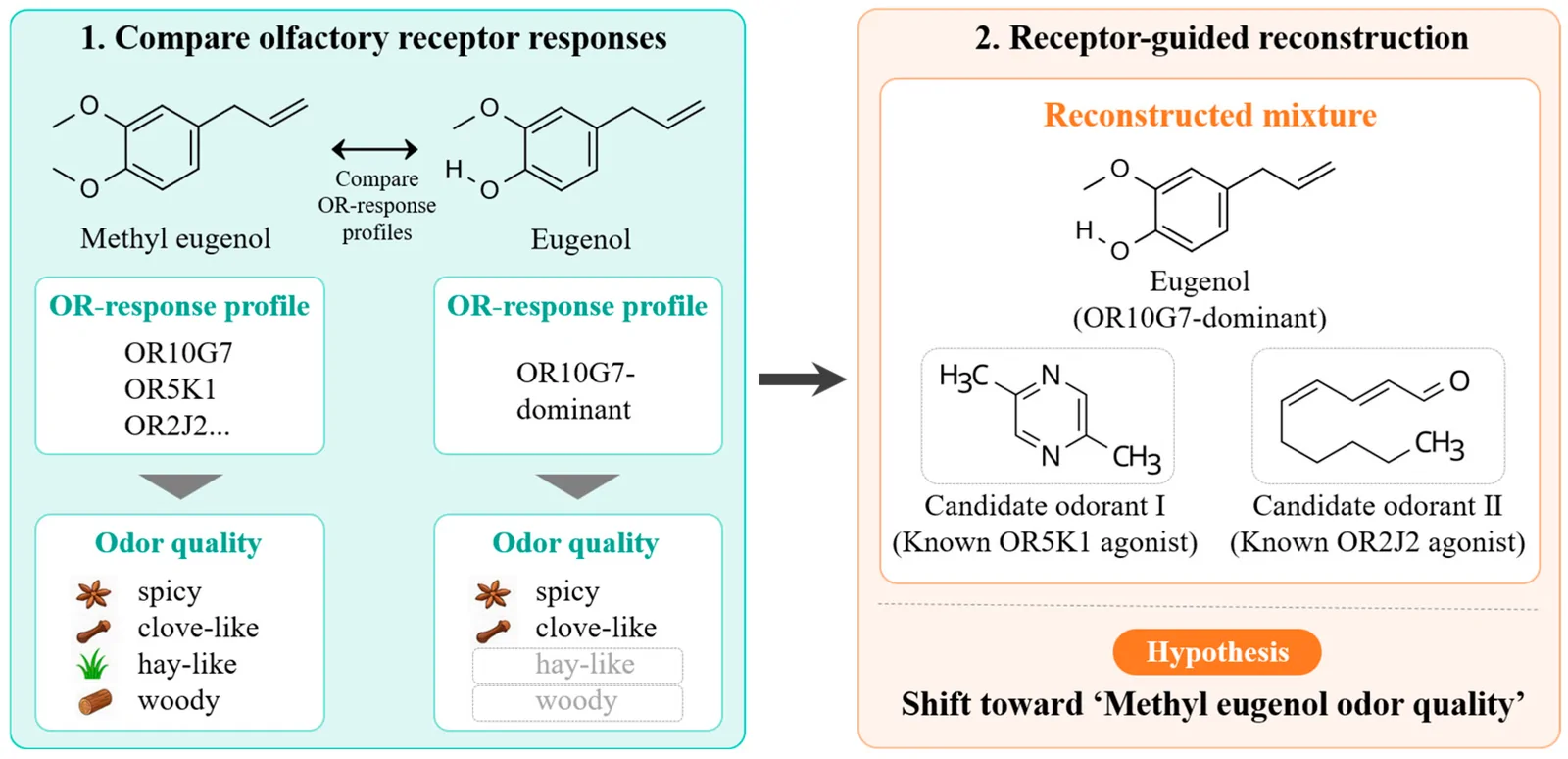
Research concept for receptor-guided candidate selection and sensory design of a mixture intended to approximate methyl eugenol odor quality. The schematic illustrates the comparison of olfactory receptor response profiles and odor characteristics between methyl eugenol and eugenol, followed by the design of the reconstructed mixture (Mix) using complementary odorants.

Based on these receptor-response differences, we selected 2,5-dimethylpyrazine (DP) and (*E*,*E*)-2,4-decadienal (Dec) as auxiliary components and combined them with eugenol to formulate a reconstructed mixture (Mix). This proof-of-concept study examined whether receptor-response differences could guide candidate selection and whether sensory evaluation and headspace analysis could characterize the resulting mixture (Figure 1). Perceptual similarity was evaluated using two-alternative forced-choice (2-AFC) tests and specific odor attributes were analyzed using Napping and check-all-that-apply (CATA) methods, respectively. Furthermore, we characterized the OR response profiles and headspace composition of the samples to evaluate the utility and limitations of the receptor-guided prediction.

## 2. Materials and Methods

### 2.1. Chemicals and Materials

The main odorant compounds used in this study were methyl eugenol, eugenol, 2,5-dimethylpyrazine, (*E*,*E*)-2,4-decadienal, methyl isoeugenol, and isoeugenol. The details of the odorant compounds are shown in Table S1, and the details of the reagents, expression vectors, and related materials used for cell culture, olfactory receptor assays, transfection, and DHS/GC–MS analysis are shown in Table S2.

### 2.2. Olfactory Receptor Expression Constructs

Human olfactory receptor genes were amplified by PCR using Human Genomic DNA: Human Mixed (Promega, Madison, WI, USA) as the template based on sequences registered in GenBank. The obtained OR coding sequences were prepared as expression constructs with a 20-amino-acid sequence derived from bovine rhodopsin added to the N-terminus (Rho-tag) [21,22]. OR10G7 (RefSeq accession: NM_001004463) and OR5K1 (RefSeq accession: NM_001004736) were cloned into the pHEK293 ultra expression vector I (Takara Bio, Shiga, Japan), and OR2J2 (RefSeq accession: NM_030905) was cloned into the pCI vector (Promega). The sequence of each expression construct was confirmed by Sanger sequencing. The corresponding empty vector was used as a control in all experiments.

### 2.3. Cell Culture and Transfection

Hana3A cells were cultured according to a system widely used for heterologous expression of mammalian olfactory receptors in HEK293T-derived cells [6,22]. Cells were maintained in D-MEM supplemented with 10% fetal bovine serum (FBS), penicillin-streptomycin, and amphotericin B. Cells were seeded in poly D-lysine-coated white 96-well plates at a density that allowed them to reach confluence after 48 h, with 50 μL of the cell suspension dispensed into each well. The cells were cultured at 37 °C in 5% CO_2_.

At 24 h after seeding, transfection was performed using FuGene 4K Transfection Reagent (Promega). The transfection mixture per well consisted of 80 ng olfactory receptor expression vector, 5 ng RTP1s vector, 10 ng pGloSensor-22F (Promega), and 0.2 μL FuGene 4K. RTP1s is known as an accessory factor that promotes cell surface trafficking and functional expression of olfactory receptors [21,23]. These components were mixed in Opti-MEM and incubated for 15 min at room temperature to prepare the transfection solution. The transfection solution was added to fresh medium and applied to the cells such that the final volume in each well was 50 μL. After transfection, the cells were cultured for an additional 24 h at 37 °C under 5% CO_2_.

### 2.4. Olfactory Receptor Functional Assay (GloSensor)

Olfactory receptor responses were measured using the GloSensor cAMP assay (Promega) according to previously reported heterologous OR response assay systems [6,22,24]. After 24 h of transfection, the culture medium was removed, and 25 μL of HBSS(+) medium containing 10 mM HEPES, 1 mM glucose, 0.75 mM CuCl_2_, and GloSensor cAMP reagent was added to each well. The plate was allowed to stand for 2 h at room temperature under light-shielded conditions to equilibrate the solution.

Odorant compounds were dissolved in DMSO to prepare stock solutions, which were diluted to the test concentrations with the HBSS(+) medium. The final DMSO concentration was maintained at approximately 0.2% (*v*/*v*) across the concentration series. After equilibration, 25 μL of the diluted odorant solution was added to each well. Firefly luciferase-derived luminescence immediately after stimulation was measured using a GloMax Discovery (Promega) at 60 s intervals for 30 cycles.

Response intensity was calculated as the area under the curve (AUC) by first normalizing the luminescence value at each cycle to that of cells transfected with the corresponding empty vector and then integrating over all 30 cycles. In the figures, the normalized AUC is represented by the normalized response. Olfactory receptor response assays were conducted in independent experiments on at least two separate days. The data shown in this study are from one experimental day, with each condition measured in three technical replicate wells.

### 2.5. Preparation of Odor Samples

Samples for sensory evaluation and DHS analysis were prepared. Each compound was prepared as a stock solution at the designated concentration, and the mixture comprised eugenol, 2,5-dimethylpyrazine, and (*E*,*E*)-2,4-decadienal. The final formulation was determined through preliminary sensory adjustment conducted separately from the formal sensory evaluations. Starting with eugenol, multiple proportions of DP and Dec were compared stepwise. The formulation judged to most closely approximate the odor quality of methyl eugenol was selected while ensuring that the characteristic aldehydic odor of Dec did not dominate the mixture.

The composition of the final formulation, Mix, is shown in Table 1. The composition of the Mix stock solution used for the olfactory receptor assay is shown in Table S6, and the concentration conditions used for the preliminary VAS and the initial main test are shown in Table S7. Because the containers and presentation or collection conditions differed between samples for sensory evaluation and samples for DHS analysis, the details are described in the relevant sections of this paper.

### 2.6. Sensory Evaluation

Sensory evaluation consisted of a VAS for intensity adjustment, two-alternative forced choice (2-AFC), and distance evaluation to confirm the similarity of the final formulation, and Napping and CATA to evaluate the odor character space and changes in attribute terms [25,26,27]. Sensory evaluation data were acquired using the FIZZ software (Acquisition ver. 2.82a03, Biosystèmes, Couternon, France), and data processing was performed using FIZZ Calculations (ver. 2.82a06, Biosystèmes, Couternon, France).

#### 2.6.1. Panelists

A panel of male and female participants aged 24–54 years participated in the sensory evaluations. The participants consisted of flavorists and researchers engaged in basic flavor research who routinely conducted flavor evaluations and flavor development. Participants were recruited internally through an open invitation and participated voluntarily after providing informed consent. No formal olfactory-function test was conducted specifically for this study, and no participants were excluded from the analyses. The number of participants differed among the tests, and the number of participants included in the analysis is indicated for each test. Some participants involved in the preliminary formulation adjustment also participated in the Napping, CATA, and final VAS evaluations.

#### 2.6.2. Common Sample Presentation Conditions

Samples for sensory evaluation were prepared in approximately 130 mL tasting glasses. Each sample was prepared by adding 30 μL of an ethanol stock solution to 30 mL of deionized water and equilibrating it for 1 h at room temperature with the lid closed. The samples were presented at room temperature. The presentation order of the samples was randomized for all sensory evaluations, and three-digit random codes were used for sample blinding.

#### 2.6.3. Odor Intensity Matching

A 0–100 mm VAS was used for intensity evaluation, with the left end labeled “much weaker” and the right end labeled “no stronger odor exists” [25]. The same scale was used for both preliminary and final VAS measurements. In the preliminary VAS, a methyl eugenol reference sample was first presented, and its intensity was standardized to 50 mm. Each sample was presented in a random order, and the relative intensities were evaluated. Eight participants were included in the preliminary VAS analyses. Based on the obtained results, the concentration for the initial main test was set, and after the initial main test, the final presentation conditions were determined using a point correction. In the final VAS, the relative intensity was evaluated using methyl eugenol as a reference (=50). Twenty-six participants (16 men and 10 women) were included in the final VAS analysis. The final concentrations of each sample are presented in Table S7.

#### 2.6.4. 2-AFC and Distance Evaluation

The similarity of the final formulation Mix to the methyl eugenol reference was evaluated using 2-AFC and distance evaluation tests [25]. In each trial, methyl eugenol was presented as the “reference”, followed by two samples labeled with three-digit codes in a random order. Participants first selected, using 2-AFC, which of the two samples was closer to the reference. Subsequently, within the same trial, the distance of each sample from the reference was evaluated on a 0–100 mm scale. The evaluation was conducted in the order of 2-AFC, followed by a distance evaluation. Six participants completed two identical evaluation sessions on separate days. Thus, 12 panelist-session observations were obtained. Because the six panelists participated in both sessions, these observations were not fully independent. These assessments were preliminary and exploratory, and no a priori power or sample-size calculation was performed.

#### 2.6.5. Napping and CATA

Napping is a rapid sensory evaluation method in which the overall perceptual distances among samples are arranged in two-dimensional space [26]. In this study, Napping was performed using the FIZZ 3.0 sensory evaluation software. The participants freely placed the presented samples in two-dimensional space. Twenty-six participants (16 men and 10 women) were included in the Napping analysis.

CATA is widely used to select perceived characteristics from multiple attribute terms [27]. In this study, CATA was conducted on a different day from Napping, and responses were collected in a multiple-selection format. Twenty-seven participants (15 men and 12 women) completed the CATA, 24 of whom also participated in the Napping and final VAS evaluations. The CATA attribute list consisted of 54 attributes, and the full list is provided in Figure S2 in the Supporting Information. For visualization, each CATA attribute was projected onto the Napping/GPA space by calculating the weighted mean of the GPA consensus coordinates of the seven samples, with the selection frequency of that attribute for each sample as the weight.

### 2.7. Dynamic Headspace Analysis

Samples for DHS analysis were prepared by adding 30 mL of deionized water to a 100 mL Dewar flask and then adding the designated amount of sample. Volatile compounds released from the prepared samples were collected using a Tenax TA-packed tube (4 mm inner diameter). The combination of DHS concentration using Tenax TA and thermal desorption–GC–MS is widely used for the collection and analysis of volatile aroma compounds [28]. The collection flow rate was 0.2 L/min, collection time was 30 min, and total collection volume was 6 L. After collection, 1 μL of 4-octanol (100 ng/μL) was added as an internal standard to each sample. The collection tube was thermally desorbed using a Gerstel thermal desorption unit (GERSTEL GmbH & Co. KG, Mülheim an der Ruhr, Germany) and introduced into the GC–MS system. GC–MS analysis was performed using an Agilent Technologies 7890A GC coupled to an Agilent Technologies 5975C Inert XL MS (Agilent Technologies, Santa Clara, CA, USA). A DB-WAX column (60 m × 0.25 mm i.d. × 0.25 μm) (Agilent Technologies) was used. The oven temperature program was as follows: holding at 50 °C for 2 min, increasing to 220 °C at 3 °C/min, and holding for 42 min. The MS was operated in SIM/Scan mode, and m/z 30–300 was recorded.

Eugenol, 2,5-dimethylpyrazine, and (*E*,*E*)-2,4-decadienal were quantified using four-point calibration curves. Each calibration curve was prepared in triplicate. The samples were measured in quadruplicate using the same prepared sample. The recovered amount was calculated as ng per DHS tube (6 L sampled) and quantified after internal standard correction.

### 2.8. Data and Statistical Analyses

Olfactory receptor response data were calculated as the AUC by first normalizing the luminescence value at each cycle to the luminescence value of the cells transfected with the corresponding empty vector and then integrating over all 30 cycles. The responses to the concentration series were displayed by connecting each of the measurement points. EC50 values for the selected receptor–odorant pairs were estimated using nonlinear regression analysis in GraphPad Prism (ver. 11.1.0, GraphPad Software, Boston, MA, USA). Excel was used for AUC calculations and the quantification of the DHS.

The Friedman test [29] was used to compare the intensity of VAS, followed by pairwise Wilcoxon signed-rank tests [30] with Holm correction [31]. The 2-AFC and distance-evaluation results were summarized descriptively, with emphasis on the consistency of the response direction across the panelist-session observations.

Generalized Procrustes Analysis (GPA) [32] was applied to the Napping data. Within each panelist, the coordinates of the two methyl eugenol presentations, methyl eugenol-1 and methyl eugenol-2, were averaged and used as methyl eugenol in the main analysis. In the GPA, translation, rotation, and isotropic scaling were performed, whereas reflections were not allowed. For the main comparisons, a paired 2D permutation test was applied to the aligned two-dimensional coordinates, and the Holm method [31] was used for multiple comparison corrections. In the main Napping comparisons, three hypothesis-driven comparisons (methyl eugenol vs. eugenol, methyl eugenol vs. Mix, and eugenol vs. Mix) were defined as the primary family, and Holm correction was applied within these three tests. Separately, all 10 pairwise comparisons among the five samples methyl eugenol, eugenol, Mix, isoeugenol, and methyl isoeugenol were analyzed as exploratory families, and Holm correction was applied within this family.

CATA data were analyzed using FIZZ Calculations (ver. 2.82a06, Biosystèmes, Couternon, France), and overall differences among samples for each attribute term were evaluated using Cochran’s Q test [33], followed by Yates-corrected McNemar pairwise comparisons when the overall test was significant. No multiplicity adjustment was applied across the 54 attributes; therefore, the CATA analyses were interpreted as exploratory and descriptive. The significance level was set at 5%. DHS data were quantified as the recovered amounts after internal standard correction and are presented as the mean ± SD of four measurements from the same prepared sample.

GPA analysis and permutation tests for Napping were performed using Python (ver. 3.11.15, Python Software Foundation, Beaverton, OR, USA). An overview of the analysis environment is provided in Supplementary Note S1.

## 3. Results and Discussion

### 3.1. Receptor-Guided Selection of Candidate Components and Sensory Design of the Mixture

We first compared the OR response profiles of methyl eugenol and eugenol to establish a receptor-level basis for designing the reconstructed mixture (Mix). Odor quality is thought to be represented, at least partly, by combinatorial patterns of activity across multiple ORs [7]. Therefore, we reasoned that differences in receptor responses between a target odorant and a structurally related base component could guide the selection of auxiliary compounds that complement the responses that are absent or weaker than those of the base components.

Eugenol was selected as the base component because of its structural similarity to methyl eugenol and their shared spicy and clove-like odor characteristics [16,17,18]. Candidate auxiliary compounds were then sought to complement the OR5K1 and OR2J2 responses, which were absent or weaker, respectively, for eugenol than for methyl eugenol. Among the pyrazines capable of activating OR5K1 and unsaturated aldehydes capable of activating OR2J2, 2,5-dimethylpyrazine (DP) and (*E*,*E*)-2,4-decadienal (Dec), respectively, were selected through preliminary sensory evaluation because they produced odor impressions that were relatively compatible with the odor character of methyl eugenol. DP and Dec were subsequently combined with eugenol to generate Mix (Figure 1), and the final formulation ratio was optimized by sensory evaluation to approximate the odor characteristics of methyl eugenol.

**Figure 2:**
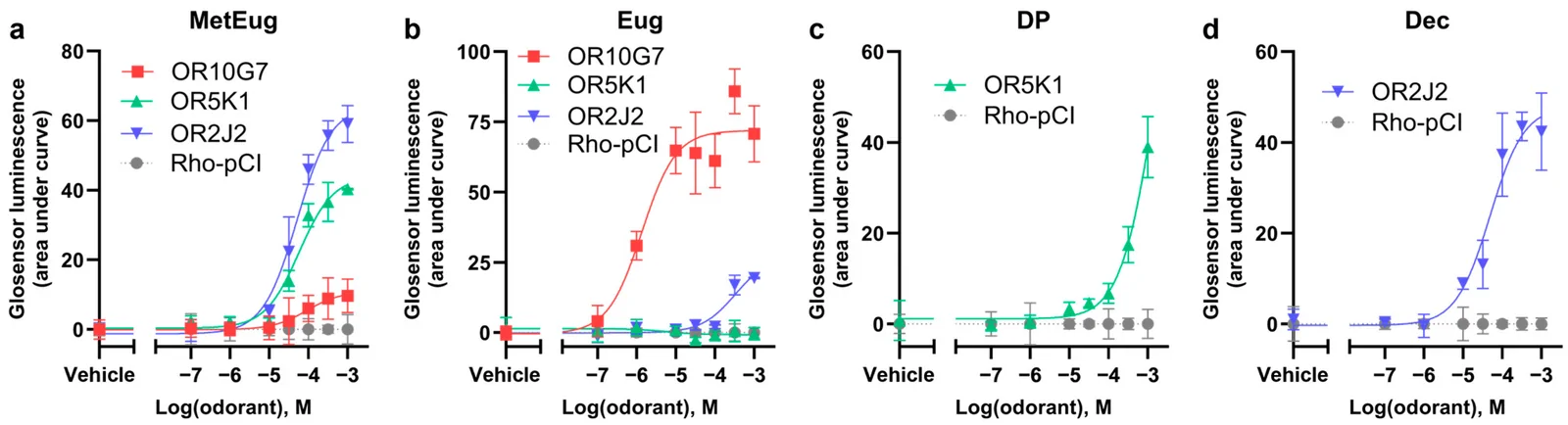
Dose–response curves of olfactory receptors for methyl eugenol, eugenol, 2,5-dimethylpyrazine, and (*E*,*E*)-2,4-decadienal. (**a**) Responses of OR10G7, OR5K1, OR2J2, and vector control to methyl eugenol. (**b**) Responses of OR10G7, OR5K1, OR2J2, and vector control to eugenol. (**c**) Responses of OR5K1 and vector control to 2,5-dimethylpyrazine. (**d**) Responses of OR2J2 and the vector control to (*E*,*E*)-2,4-decadienal. The horizontal axis indicates the odorant concentration [log(M)], and the vertical axis indicates the normalized response. Data are shown as the mean ± SD of three wells measured on the same day under the same conditions. MetEug: methyl eugenol; Eug: eugenol; DP: 2,5-dimethylpyrazine; Dec: (*E*,*E*)-2,4-decadienal.

Methyl eugenol elicited concentration-dependent responses from OR10G7, OR5K1, and OR2J2 [19,20]. In contrast, eugenol showed a strong response to OR10G7 but showed little activity toward OR5K1 and weaker activity toward OR2J2 than methyl eugenol. 2,5-dimethylpyrazine predominantly activated OR5K1, whereas (*E*,*E*)-2,4-decadienal activated OR2J2 (Figure 2). OR5K1 recognizes pyrazine compounds (alkylpyrazines) with roasted-like odors [34], whereas OR2J2 responds to unsaturated aldehydes, including (*E*)-2-nonenal [35]. Thus, the responses of OR5K1 to 2,5-dimethylpyrazine and OR2J2 to (*E*,*E*)-2,4-decadienal observed in this study were consistent with previously reported ligand specificities of these receptors.

These results demonstrate that the distinction between methyl eugenol and eugenol can be described not only in terms of their chemical structures and perceptual characteristics but also by their OR response profiles. Specifically, compared with methyl eugenol, eugenol lacked substantial OR5K1 activity and exhibited weaker OR2J2 activity. 2,5-dimethylpyrazine and (*E*,*E*)-2,4-decadienal were selected as auxiliary components capable of complementing these response differences. Accordingly, Mix was designed using a two-stage strategy, in which receptor-response differences were first used to identify candidate compound classes, followed by preliminary sensory evaluation to select individual auxiliary components and optimize their formulation.

Concentration–response curves were analyzed using nonlinear regression. Well-defined curve fits (R^2^ = 0.88–0.95; Table S4) were obtained for the (*E*,*E*)-2,4-decadienal–OR2J2, eugenol–OR2J2, and methyl eugenol–OR5K1 combinations. The methyl eugenol–OR10G7 fit had a comparatively lower R^2^ (0.54); therefore, its EC_50_ estimate should be interpreted cautiously. For some receptor-odorant combinations, the tested concentration range did not reach the response plateau. Consequently, the upper asymptote of the dose–response curve could not be reliably defined, and a reliable EC_50_ could not be estimated. For DP–OR5K1, although nonlinear regression yielded a fit with R^2^ > 0.9, a measurable response was observed primarily at the highest concentration tested, and the upper asymptote (TOP) was not reliably estimated. The EC_50_ value shown in Table S4 (1.47 mM) is therefore extrapolated and should be regarded as indicative rather than as a reliable potency estimate. The response observed mainly at high concentrations is consistent with a previous study reporting an EC_50_ of approximately 2.2 mM for OR5K1 activation by 2,5-dimethylpyrazine [34]. Therefore, we focused primarily on the presence and concentration dependence of receptor activation rather than on direct comparisons of potency.

Previously reported OR responses, odor descriptors, and odor thresholds for methyl eugenol, eugenol, 2,5-dimethylpyrazine, and (*E*,*E*)-2,4-decadienal are summarized in the Supporting Information (Tables S3 and S5). Methyl eugenol and eugenol share spicy and clove-like descriptors, whereas methyl eugenol has also been described as hay-like, dried-grass, and woody [16,17,18]. Although the two compounds belong to the same general category, they possess distinct perceptual characteristics. In contrast, 2,5-dimethylpyrazine is primarily described using roasted-type terms, whereas (*E*,*E*)-2,4-decadienal is commonly associated with fatty and green-type descriptors [36]. Furthermore, their odor thresholds differ substantially, with (*E*,*E*)-2,4-decadienal exhibiting a particularly low threshold [37,38].

Consequently, the selection of 2,5-dimethylpyrazine and (*E*,*E*)-2,4-decadienal could not be predicted from the similarities in conventional odor descriptors alone. Instead, the differences between the OR response profiles of methyl eugenol and eugenol provided a mechanistic basis for narrowing down the candidate compound classes, whereas preliminary sensory evaluation enabled the selection of compatible compounds within those classes. Thus, the novelty of this approach lies in using receptor-response differences as the starting point for candidate selection and integrating this information with sensory evaluation to construct a mixture that approximates the target odor.

### 3.2. Presentation Intensity Adjustment and Preliminary Evaluation of the Mix

Because Mix was designed based on OR response differences and subsequently optimized through sensory evaluation, we determined the presentation conditions for subsequent sensory evaluation tests. Perceived odor quality can vary substantially with odor intensity [39], and matching sample intensities as closely as possible (i.e., establishing approximately iso-intense conditions) is important for evaluating differences in odor quality, independently of intensity [25]. Therefore, we conducted a preliminary visual analog scale (VAS) assessment to determine the presentation concentration that produced comparable perceived intensities across samples.

In the preliminary VAS assessment, methyl eugenol was used as a reference and assigned an intensity value of 50. The relative intensity of each sample was evaluated at three concentrations and dilution levels. Based on the resulting concentration–intensity relationships, the concentration or dilution level expected to produce an intensity closest to that of the methyl eugenol reference was estimated for each sample and used in the initial main test. Because residual differences in perceived intensity were observed in this test, the presentation conditions were further adjusted once based on the mean VAS scores. The concentration–intensity relationships obtained from the preliminary VAS are shown in Figure S1, and the presentation conditions used in the preliminary VAS and the initial main test are summarized in Table S7.

**Figure 3:**
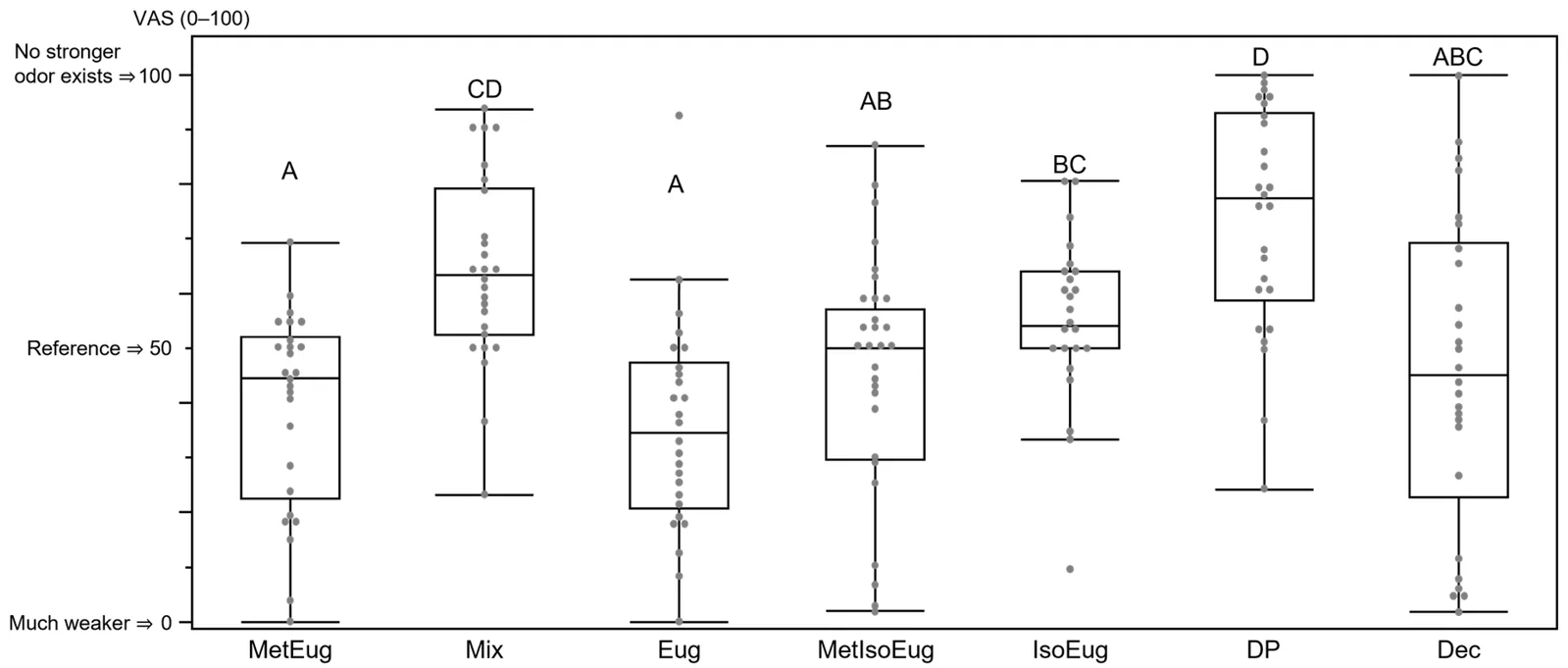
Evaluation of iso-intensity conditions using the intensity VAS. Each sample was presented under aqueous headspace conditions, and the relative intensity of each sample was evaluated using VAS, with methyl eugenol as the reference (=50). Box plots show the distribution of each sample, and the colored dots represent individual panelist ratings, with colors corresponding to the respective samples. The indicators on the left denote the VAS anchor points at 0 (“much weaker”), 50 (reference), and 100 (“no stronger odor exists”). The analysis included 26 panelists. Letter groups indicate groupings based on pairwise Wilcoxon signed-rank tests with Holm correction after the Friedman test; groups sharing the same letter are not significantly different from each other. MetEug: methyl eugenol; Eug: eugenol; MetIsoEug: methyl isoeugenol; IsoEug: isoeugenol; DP: 2,5-dimethylpyrazine; Dec: (*E*,*E*)-2,4-decadienal.

Under readjusted presentation conditions, the perceived intensity of each sample was evaluated using an intensity VAS. Although complete iso-intensity was not achieved, the intensities of methyl eugenol, methyl isoeugenol, and eugenol fell within a relatively similar range (Figure 3). In contrast, 2,5-dimethylpyrazine and Mix were perceived as significantly more intense than methyl eugenol and eugenol (Figure 3). Nevertheless, the adjusted conditions reduced large differences in perceived intensity while avoiding the use of excessively high concentrations that could promote olfactory fatigue or alter the odor quality. These conditions were used in subsequent odor quality evaluations. Because Mix and 2,5-dimethylpyrazine remained more intense than methyl eugenol and eugenol, the residual intensity differences were considered when interpreting the results (Figure 3).

**Figure 4:**
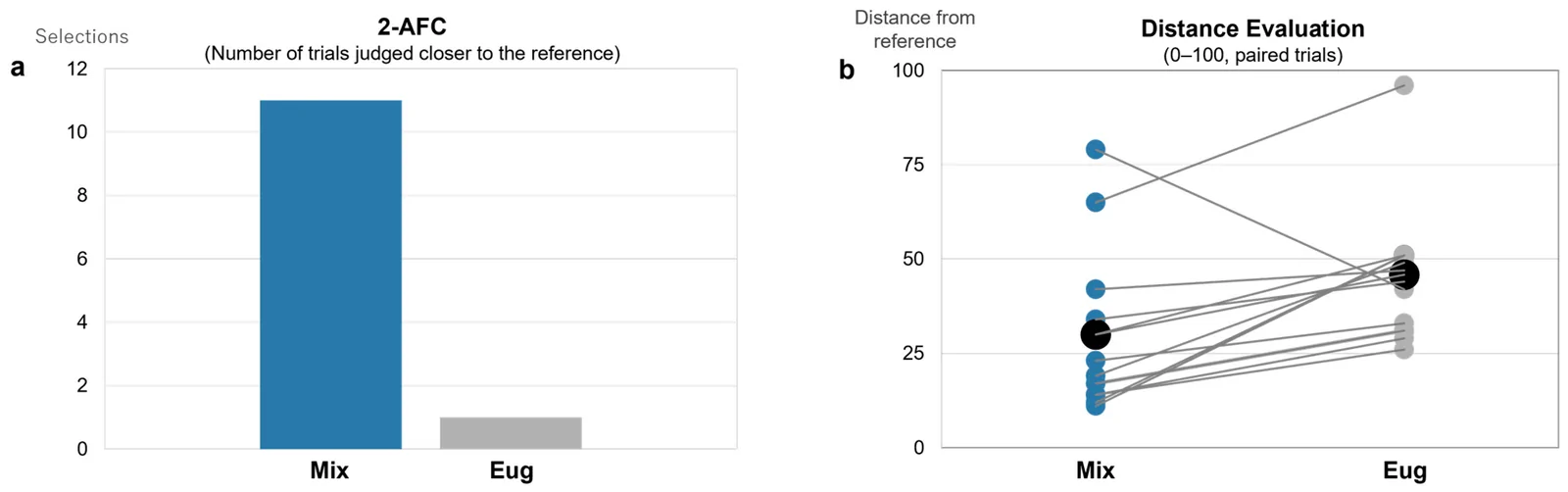
Similarity evaluation of the final formulation Mix by 2-AFC and distance evaluation using methyl eugenol as the reference. (**a**) Methyl eugenol was used as the reference sample, and the participants evaluated the samples using 2-AFC to determine whether Mix or eugenol was closer to the reference sample. (**b**) Distances of Mix and eugenol from the reference were evaluated within the same trial. The colored dots represent individual trial-level evaluations, with colors corresponding to Mix and Eug. Each line connects the paired data obtained in the same trial. The data were based on 12 trials from six panelists who participated in two sessions. Eug: eugenol.

In the 12 panelist-session observations, Mix was selected as more similar to methyl eugenol than eugenol in 11 observations in the 2-AFC test. The paired distance evaluation showed the same directional pattern, with Mix rated as closer to the reference in 11 of the 12 observations (Figure 4). These descriptive results show a similar directional pattern in the two evaluations.

However, because this study involved six panelists who each participated in two sessions on separate days, the two observations from each panelist cannot be assumed to be fully independent. Therefore, these results should be interpreted as preliminary and exploratory evidence of response consistency rather than as confirmatory statistical evidence.

### 3.3. Characterization of the Odor Quality Shift of Mix Using Sensory Space and Attribute Analyses

Under the adjusted presentation conditions, we characterized the sensory position and odor attributes of Mix using Napping and CATA. Napping, also known as projective mapping, is a method for arranging perceptual relationships among samples in a two-dimensional space [26], whereas CATA provides an attribute-based characterization of sensory profiles [27]. By combining these approaches, we evaluated whether the odor character of the Mix shifted from that of eugenol to that of methyl eugenol.

**Figure 5:**
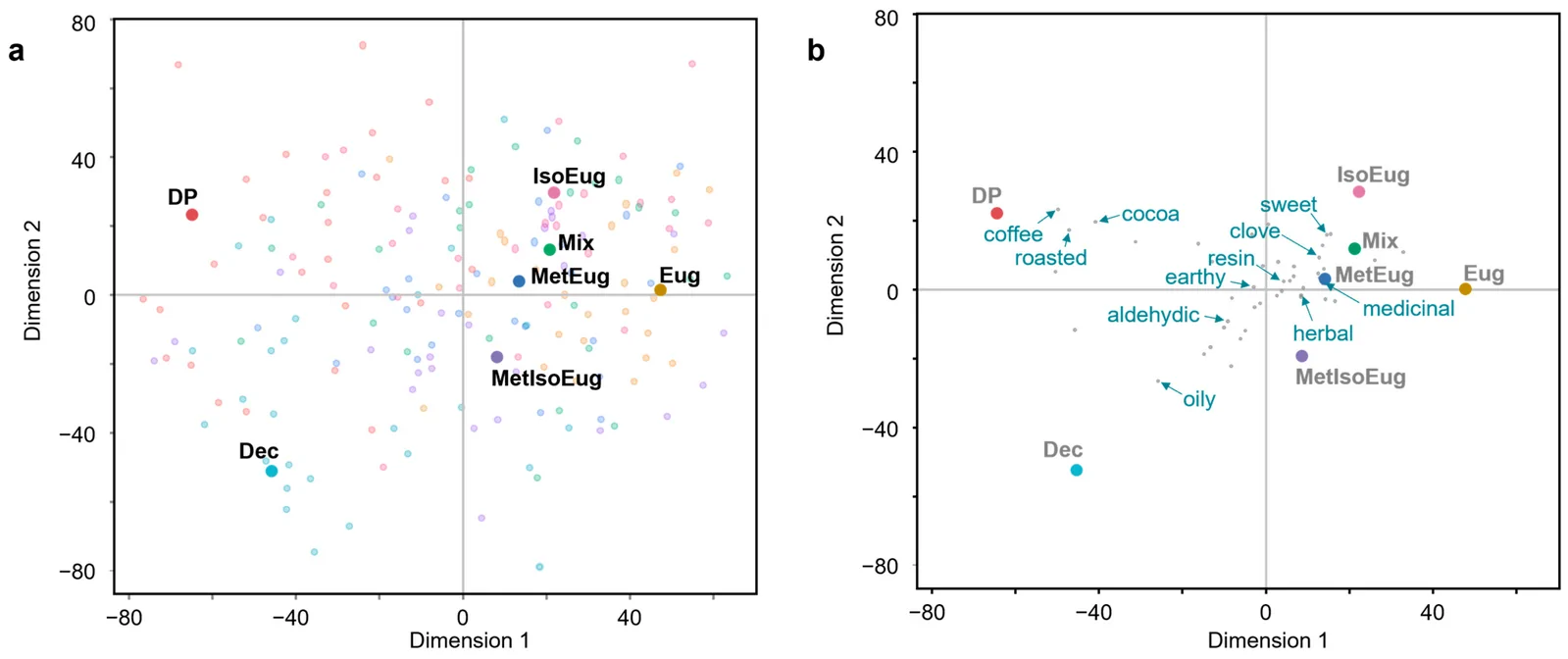
Odor character shift of Mix shown by Napping and GPA-based CATA integration. (**a**) GPA consensus map obtained from Napping of the seven samples. (**b**) CATA attribute terms projected onto the same GPA space. The colored dots represent the seven samples, with colors used consistently to distinguish the samples across both panels. The projected attribute terms indicate the sensory characteristics related to the relative positions of the samples. MetEug: methyl eugenol; Eug: eugenol; MetIsoEug: methyl isoeugenol; IsoEug: isoeugenol; DP: 2,5-dimethylpyrazine; Dec: (*E*,*E*)-2,4-decadienal.

In the consensus Napping configuration obtained by generalized Procrustes analysis (GPA), Mix was positioned away from eugenol and generally shifted toward methyl eugenol (Figure 5a). However, Mix did not completely overlap with methyl eugenol and occupied an intermediate region that extended toward isoeugenol. Thus, the sensory position of Mix shifted from that of eugenol toward methyl eugenol, but the odor quality of methyl eugenol was not completely reproduced.

The projection of the CATA attributes onto the GPA sensory space showed that clove, sweet, medicinal, herbal, and resin were associated with the region surrounding Mix, whereas aldehydic was positioned away from the Mix (Figure 5b). Moreover, attributes associated with the individual odor qualities of the auxiliary components 2,5-dimethylpyrazine and (*E*,*E*)-2,4-decadienal, including oily, roasted, coffee, and cocoa, were not positioned near Mix. This distribution suggests that the odor quality of Mix was not perceived as the sum of the characteristic odors of its individual auxiliary components.

**Figure 6:**
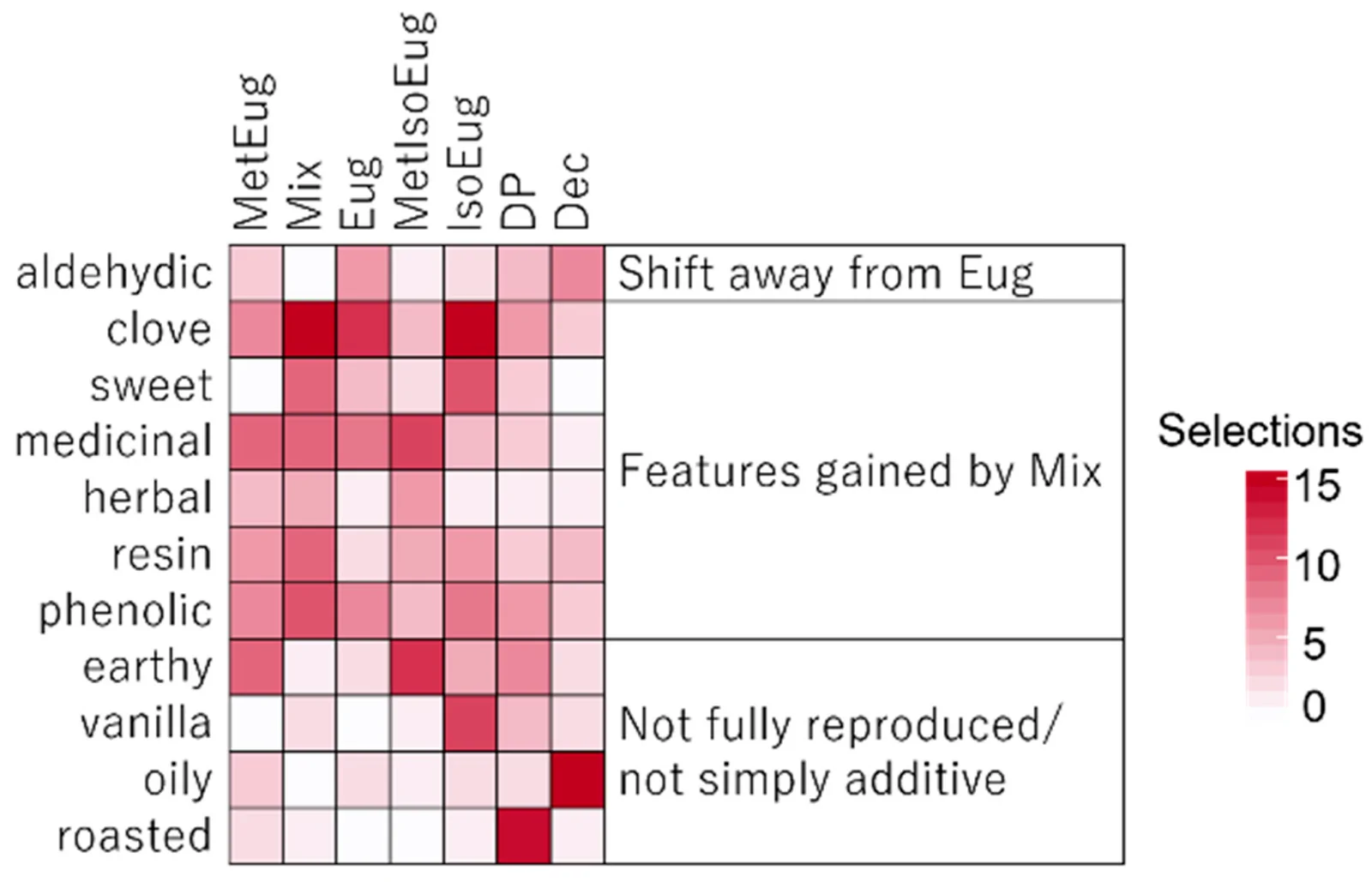
CATA selection-count heatmap for representative odor attributes selected for descriptive interpretation of the positional shift of Mix. The color of each cell indicates the number of selections for the corresponding attribute term, with darker colors representing higher selection counts (*n* = 27). The columns were arranged with methyl eugenol, Mix, and eugenol placed first for the primary comparison, followed by methyl isoeugenol, isoeugenol, and the auxiliary components 2,5-dimethylpyrazine and (*E*,*E*)-2,4-decadienal. In the Mix, the aldehydic notes were lower, and clove and sweet notes were higher than those in eugenol. Medicinal was close to the level of methyl eugenol, and herbal, resin, and phenolic were also higher than those of eugenol alone. In contrast, earthy was low, suggesting that Mix did not fully reproduce the characteristics of methyl eugenol. Furthermore, oily derived from (*E*,*E*)-2,4-decadienal and roasted derived from 2,5-dimethylpyrazine were selected at high frequencies for the single components but were not apparent in the Mix. MetEug: methyl eugenol; Eug: eugenol; MetIsoEug: methyl isoeugenol; IsoEug: isoeugenol; DP: 2,5-dimethylpyrazine; Dec: (*E*,*E*)-2,4-decadienal.

The CATA heatmap further showed that the attribute profile of Mix differed from that of eugenol (Figure 6; Table S9). The aldehydic attribute was selected for eugenol but not for Mix, whereas clove and sweet were selected more frequently for Mix than for eugenol. Medicinal was selected at similar frequencies for Mix and methyl eugenol, and herbal, resin, and phenolic were also selected more frequently for Mix than for eugenol. In contrast, earthy was selected less frequently for Mix than for methyl eugenol. Collectively, these patterns indicate that Mix acquired several attributes associated with methyl eugenol while failing to reproduce its complete profile.

The absence of prominent oily, roasted, coffee, and cocoa attributes in Mix is consistent with configural perception, in which the components of an odor mixture are integrated into a new odor object rather than being perceived independently as a simple elemental combination [40]. Accordingly, the sensory change produced by adding 2,5-dimethylpyrazine and (*E*,*E*)-2,4-decadienal to eugenol appears to reflect the emergence of an integrated odor quality rather than the direct addition of characteristic odors of auxiliary components.

**Figure 7:**
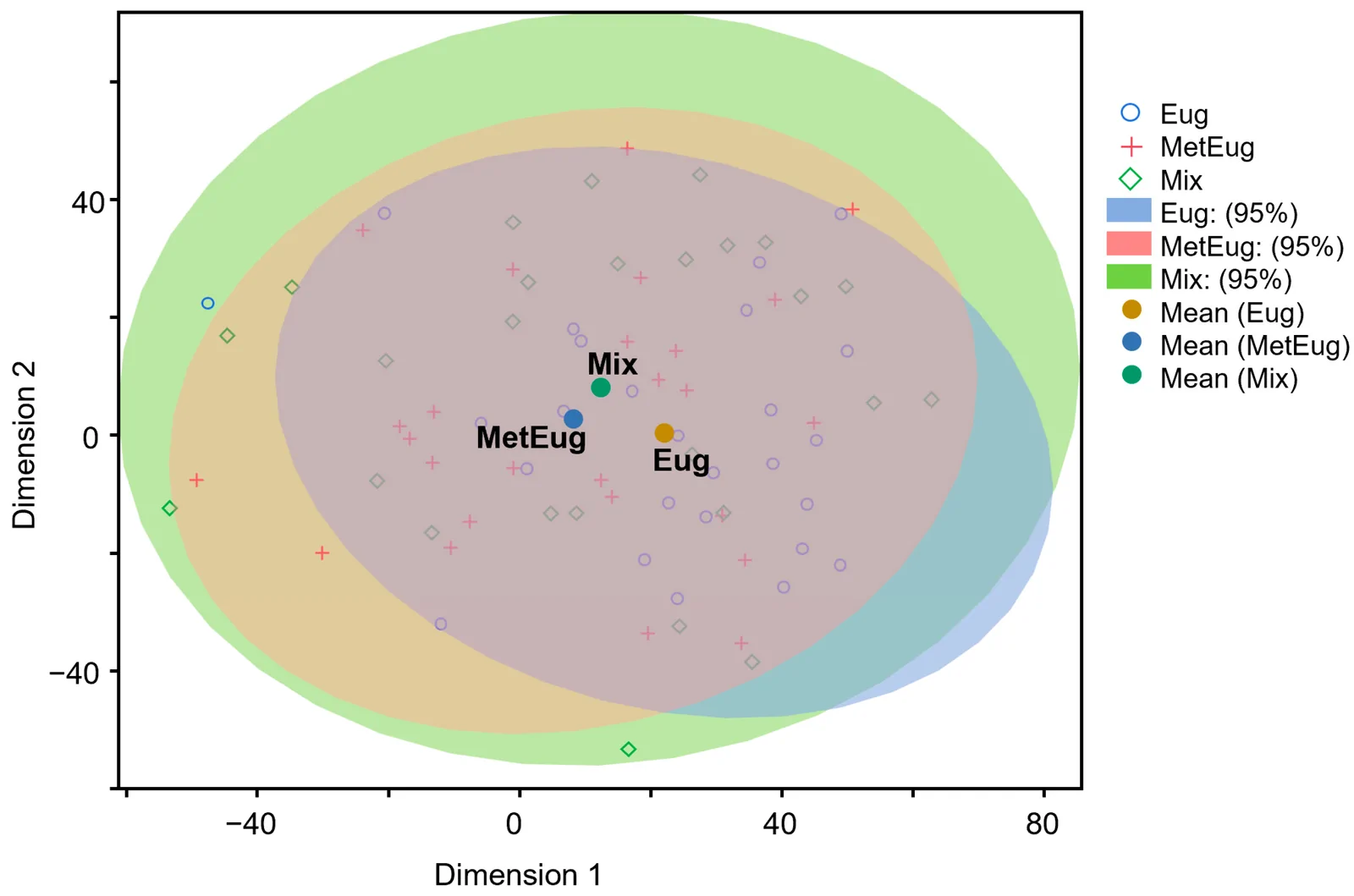
Distribution of methyl eugenol, eugenol, and Mix in the Napping sensory space. Napping data were aligned by GPA without reflection, and the coordinate distributions across the panelists for methyl eugenol, eugenol, and Mix are shown as ellipses. The ellipses indicate the panel-to-panel variability for each sample (95% coverage), and the center points indicate the mean positions. MetEug: methyl eugenol; Eug: eugenol.

Visualization of the sample distributions using ellipses likewise showed that the centroid of Mix was separated from that of eugenol and positioned between eugenol and methyl eugenol (Figure 7). Based on the coordinate distances, the centroid of Mix was closer to methyl eugenol than to eugenol, although it did not coincide with methyl eugenol. This spatial pattern is consistent with a descriptive directional tendency for Mix to lie between eugenol and methyl eugenol, rather than indicating complete reconstruction of the target odor quality.

In paired two-dimensional permutation tests for the three primary comparisons, methyl eugenol and eugenol differed significantly in sensory position after adjustment for multiple comparisons (Holm-adjusted *p* = 0.043), confirming that their odor qualities were distinguishable under the present experimental conditions. No significant difference was detected between methyl eugenol and Mix (Holm-adjusted *p* = 0.704). However, because the absence of a significant difference does not establish sensory equivalence, this result only indicates that the analysis did not detect a spatial difference between Mix and methyl eugenol. The difference between eugenol and Mix was also not statistically significant after Holm adjustment (adjusted *p* = 0.081), although the observed spatial displacement of Mix was consistent with the directional shift indicated by the descriptive Napping and CATA results (Table 2).

The receptor-guided design used in this study was limited to OR10G7, OR5K1, and OR2J2; therefore, it could not be expected to capture the complete receptor-response pattern underlying the odor quality of methyl eugenol. Although Mix acquired several methyl eugenol-associated characteristics, the earthy attribute was not sufficiently reproduced. Responses mediated by ORs not examined in this study, differences in the relative magnitudes of receptor activation, or receptor-level interactions specific to the mixture may contribute to the remaining perceptual differences.

When the Holm correction was applied separately to the exploratory family comprising all 10 pairwise comparisons among methyl eugenol, eugenol, Mix, isoeugenol, and methyl isoeugenol, none of the pairwise differences were statistically significant (Table S10). This lack of significance may reflect both the relatively overlapping sensory distributions of the samples and the limited statistical power associated with the small panel size and the adjustment for multiple comparisons. Accordingly, the descriptive Napping configuration showed a directional pattern for Mix from eugenol toward methyl eugenol; however, the exploratory analysis did not establish statistically significant separation among the five samples. In addition, because some participants involved in the preliminary formulation adjustment also participated in the Napping, CATA, and final VAS evaluations, potential bias associated with prior exposure to the formulation process cannot be excluded.

### 3.4. Olfactory Receptor Response Profile and Headspace Characteristics of Mix

Finally, we characterized the sensory-optimized Mix in terms of its OR response profile and headspace characteristics and examined how these properties corresponded to the sensory results.

**Figure 8:**
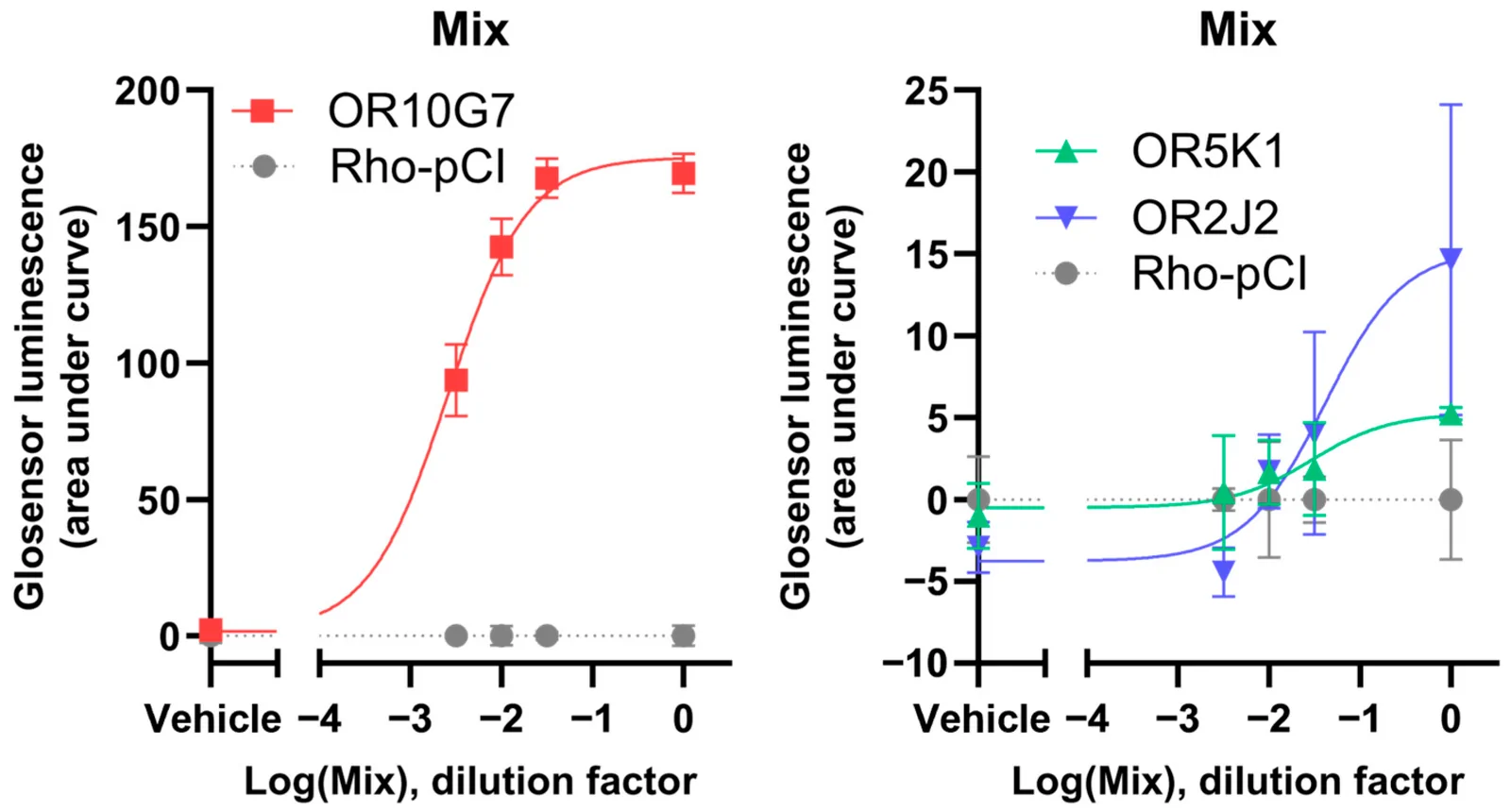
Olfactory receptor response curves of Mix under liquid-phase stimulation using a dilution series. Olfactory receptor responses are shown for the Mix presented as a dilution series (vehicle, 1/300, 1/100, 1/30, 1/10, and undiluted). OR10G7 and vector control are shown on the left, and OR5K1, OR2J2, and vector control are shown on the right. Because the response scales differed greatly between OR10G7 and OR5K1/OR2J2, the vertical axis scales are shown separately for the left and right panels. The response values are presented as normalized responses. Data are shown as the mean ± SD of three wells measured on the same day under the same plate conditions.

When responses to a dilution series of Mix were evaluated under liquid-phase stimulation, OR10G7 showed relatively strong responses over a broad dilution range. In contrast, OR5K1 showed smaller responses that were primarily observed at higher concentrations, whereas OR2J2 responded only weakly under the present assay conditions (Figure 8). These results indicate that the sensorially optimized Mix showed an OR10G7-dominant response pattern in the receptor assay under liquid-phase stimulation (Figure 8). Thus, the response profile of Mix was dominated by OR10G7. This profile did not fully reproduce that of methyl eugenol, which activated OR10G7, OR5K1, and OR2J2. Instead, Mix retained an eugenol-associated OR10G7-dominant response profile despite the directional shift from eugenol toward methyl eugenol observed in the sensory evaluations.

**Figure 9:**
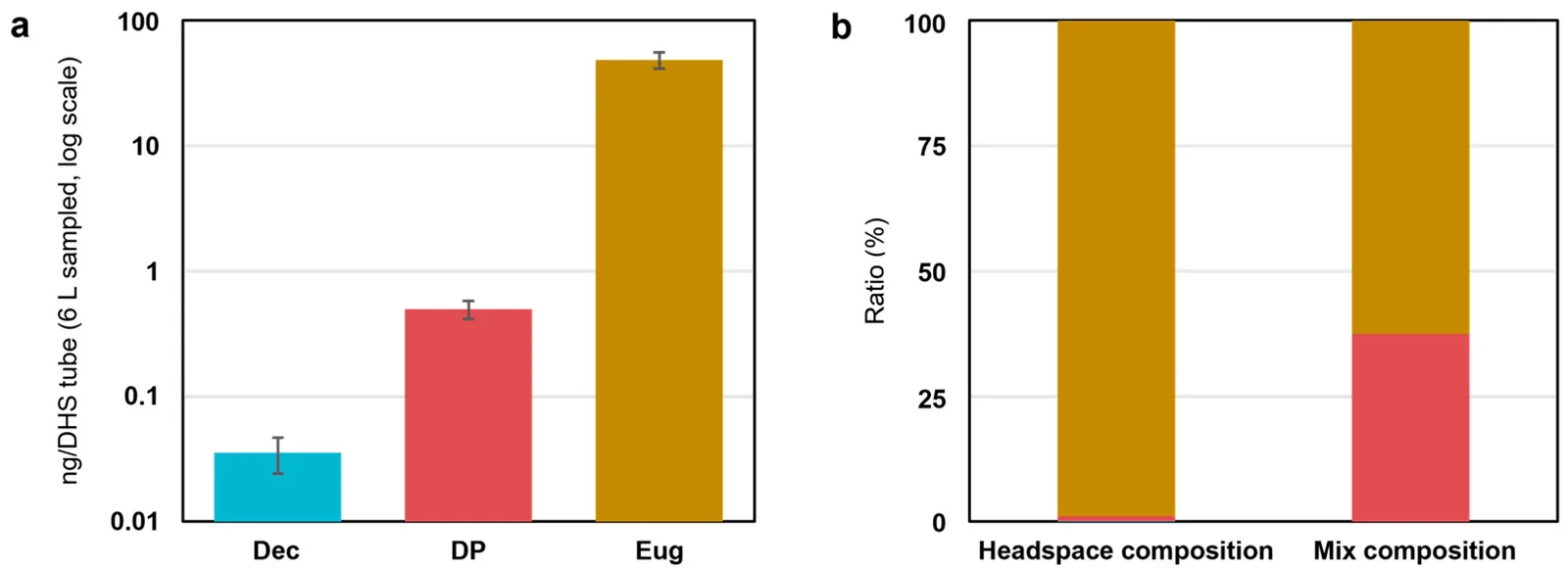
Dynamic headspace analysis of Mix. (**a**) Recovered amounts of eugenol, 2,5-dimethylpyrazine, and (*E*,*E*)-2,4-decadienal collected from the headspace of Mix by dynamic headspace sampling (DHS), shown as ng per DHS tube (6 L sampled). (**b**) Comparison between the formulation ratio (%) of Mix and the relative composition ratio (%) in DHS. The colors distinguish the three components: Dec, DP, and Eug. Dec: (*E*,*E*)-2,4-decadienal; DP: 2,5-dimethylpyrazine; Eug: eugenol.

DHS analysis showed that eugenol was the predominant volatile component detected from Mix. The relative DHS composition calculated from the quantified recovered amounts was 98.92% eugenol, 1.01% 2,5-dimethylpyrazine, and 0.07% (*E*,*E*)-2,4-decadienal (Figure 9 and Table 3). In contrast, their proportions in the liquid formulation were 62.50%, 37.50%, and 0.00021%, respectively.

Thus, the relative distribution of the components detected by DHS differed markedly from their proportions in the liquid formulation. Although 2,5-dimethylpyrazine constituted a substantial proportion of the liquid formulation, it accounted for only a small proportion of the total amount recovered by DHS. Conversely, (*E*,*E*)-2,4-decadienal was detected by DHS despite its low proportion in the liquid formulation. Because DHS recovery may differ among compounds, the relative DHS composition based on the quantified recovered amounts should not be interpreted as the absolute molar composition of the headspace gas. The higher relative proportion of Dec in the DHS-recovered fraction likely reflects compound-specific differences in liquid-to-headspace partitioning, release, and DHS collection efficiency. Nevertheless, the results indicate that the liquid formulation ratio does not directly represent the relative distribution of volatile compounds detected after their release from the samples.

Despite the OR10G7-dominant response profile and the predominance of eugenol in the DHS analysis, sensory analyses indicated that the Mix was directionally shifted away from eugenol and toward methyl eugenol. This finding suggests that 2,5-dimethylpyrazine and (*E*,*E*)-2,4-decadienal may have influenced the odor quality, even though they were recovered in relatively small amounts by DHS or produced limited responses in the liquid-phase OR assay. In particular, (*E*,*E*)-2,4-decadienal has an extremely low reported odor threshold (Table S5), suggesting that even a small amount released into the headspace can exert a perceptual effect. However, the perceptual contribution of each component cannot be quantitatively inferred from its relative DHS-recovered amount alone.

The discrepancy among the liquid formulation ratio, liquid-phase OR response profile, DHS measurements, and sensory results highlights an important limitation of receptor-guided odor reconstruction. Heterologous OR assays do not fully reproduce the native receptor abundance, cell surface expression, membrane environment, or delivery of volatile compounds to the olfactory epithelium [22,23].

Furthermore, liquid-phase stimulation does not account for compound-specific releases into the headspace. Vapor-phase OR assays, which expose receptors to volatile compounds under conditions more closely related to orthonasal odor delivery [24], may provide response profiles that are more relevant to sensory perception.

However, vapor-phase stimulation alone does not necessarily allow for the direct prediction of odor quality. Receptor responses to mixtures are not always simple sums of the responses to their individual components because competition, inhibition, and antagonism can modify the ensemble responses [11,41]. These receptor-level interactions have also been shown to influence human odor perception [13]. Therefore, the response to a mixture cannot be assumed to be equal to the concentration-weighted sum of the responses to eugenol, 2,5-dimethylpyrazine, and (*E*,*E*)-2,4-decadienal measured separately. Future analyses combining vapor-phase stimulation with direct measurement of the response to the complete mixture will be important for determining how the headspace composition of Mix is translated into its OR response pattern.

The receptor set used for the mixture design was limited to OR10G7, OR5K1, and OR2J2; therefore, it could not capture the complete receptor-response pattern underlying the odor quality of methyl eugenol. This limitation may partly explain why Mix acquired several methyl eugenol-associated characteristics but did not reproduce its complete sensory profile, including the earthy attribute of methyl eugenol. Responses mediated by ORs that were not examined in this study, differences in the relative magnitude of receptor activation, and mixture-specific receptor interactions may contribute to the remaining perceptual differences. In addition, the perception of complex odors is shaped not only by peripheral receptor activity but also by higher-order olfactory processing, in which odor identity, similarity, and quality are represented in the piriform cortex and related brain regions [42,43]. Contributions from non-OR sensory pathways and higher-order olfactory processing cannot be excluded, although these mechanisms were not directly examined in the present study.

Nevertheless, the differences in OR responses between methyl eugenol and eugenol provide a useful starting point for candidate selection. 2,5-dimethylpyrazine and (*E*,*E*)-2,4-decadienal were selected to target the receptor-response difference associated with OR5K1 and OR2J2 components of the methyl eugenol response profile that were not adequately represented by eugenol. Subsequent adjustment of their mixing ratios through sensory evaluation yielded a mixture whose odor quality showed a descriptive shift from eugenol toward methyl eugenol. However, because the final Mix retained an OR10G7-dominant response profile under the present assay conditions, the current results do not establish complete receptor-level complementation. Thus, the OR response profiles did not directly predict the final formulation or its complete odor quality but served as a mechanistic guide for narrowing the candidate space.

Overall, this proof-of-concept study suggests that olfactory receptor response differences can provide a mechanistically informed basis for candidate selection, while sensory evaluation is needed to optimize and assess the resulting mixture. Future studies using chemically diverse odorants and broader receptor sets are needed to determine the generalizability of this approach.

## Figures and Tables

**Table 1:** Composition of the final formulation Mix.

Compound	Converted Proportion (%)
eugenol	62.5
2,5-dimethylpyrazine	37.5
(*E*,*E*)-2,4-decadienal	0.00021

**Table 2:** Paired 2D permutation test results for the three primary Napping comparisons.

Sample A	Sample B	*p*	Holm-Adjusted *p*	Signif.
methyl eugenol	eugenol	0.014	0.043	*
methyl eugenol	Mix	0.704	0.704	n.s.
eugenol	Mix	0.041	0.081	n.s.

*p*: unadjusted *p*-value; Holm-adjusted *p*: *p*-value after Holm correction; Signif.: significance; *: Holm-adjusted *p* < 0.05; n.s.: not significant.

**Table 3:** DHS recovery and composition of Mix components.

Component	Recovered Amount (ng/DHS Tube)	SD	DHS Composition (%)	Mix Composition (%)
eugenol	48.495	7.432	98.92	62.50
2,5-dimethylpyrazine	0.496	0.083	1.01	37.50
(*E*,*E*)-2,4-decadienal	0.036	0.011	0.07	0.00021

The recovered amounts are shown as mean values from four measurements of the same prepared sample; SD indicates the standard deviation of the measurements.

## Data Availability

The processed data supporting the findings of this study are included in the article and its Supporting Information. Additional anonymized sensory data and the analysis code may be made available by the corresponding authors upon reasonable request, subject to institutional review and ethical, privacy, and intellectual-property considerations.

## Supplementary Materials

The following supporting information can be downloaded at https://www.mdpi.com/article/10.3390/foods15213963/s1, Table S1: Details of odorant compounds; Table S2: Reagents and materials used for cell culture, olfactory receptor assays, transfection, and DHS/GC–MS analysis; Table S3: Previously reported olfactory receptor responses and EC50 values relevant to Mix design [44]; Table S4: EC50 values estimated from concentration–response curves in this study; Table S5: Previously reported odor descriptors and odor thresholds related to Mix design; Table S6: Composition of the Mix stock solution used for sensory evaluation and olfactory receptor assays; Table S7: Mean VAS values in the initial main test and presentation concentrations before and after one-point correction; Table S8: CATA attribute-wise Cochran’s Q test results; Table S9: Selection counts for representative CATA attributes; Table S10: Matrix of Holm-adjusted *p*-values for all 10 pairwise comparisons among the five samples in Napping; Figure S1: Concentration–response relationships of each sample in the preliminary VAS; Figure S2: CATA selection-frequency heatmap for all 54 attributes; Note S1: Napping GPA analysis environment and analysis settings.

## Abbreviations

The following abbreviations are used in this manuscript:

MetEug, methyl eugenol; Eug, eugenol; DP, 2,5-dimethylpyrazine; Dec, (*E*,*E*)-2,4-decadienal; MetIsoEug, methyl isoeugenol; IsoEug, isoeugenol; OR, olfactory receptor; VAS, visual analog scale; 2-AFC, two-alternative forced choice; CATA, check-all-that-apply; GPA, Generalized Procrustes Analysis; DHS, dynamic headspace; GC–MS, gas chromatography–mass spectrometry; AUC, area under the curve.
