# Supplementary material for: Sensory Evaluation of a Receptor-Guided Mixture Designed to Approximate Methyl Eugenol Odor Quality
Source: Foods. 2026 Nov 4;15(21):3963. doi: 10.3390/foods15213963 (PMC13763400; doi:10.3390/foods15213963)
Supplement: Supplementary file 1 [file foods-15-03963-s001.zip › foods-4622633-supplementary.pdf]

Supporting Information

Sensory Validation of a Receptor-Guided Mixture Designed to Approximate Methyl Eugenol Odor Quality

Table S1. Details of odorant compounds

Reagent / material	Abbrev.	Purity (%)	Source	Identifier
methyl eugenol	MetEug	>98.0	Tokyo Chemical Industry (TCI)	Cat# D1360
eugenol	Eug	≧98.0	Sigma-Aldrich	Cat# W246700
2,5-dimethylpyrazine	DP	>98.0	Tokyo Chemical Industry (TCI)	Cat# D2171
(*E*,*E*)-2,4-decadienal	Dec	>90.0	Tokyo Chemical Industry (TCI)	Cat# D1934
methyl isoeugenol	MetIsoEug	>98.0	Tokyo Chemical Industry (TCI)	Cat# P1103
isoeugenol	IsoEug	>97.0	Tokyo Chemical Industry (TCI)	Cat# I0132
4-octanol	—	>97.0	Tokyo Chemical Industry (TCI)	Cat# O0155

Table S2. Reagents and materials used for cell culture, olfactory receptor assays, transfection, and DHS/GC-MS analysis

Reagent / material	Source	Identifier
D-MEM	FUJIFILM Wako Pure Chemical	Cat# 043-30085
fetal bovine serum (FBS)	Biowest	Cat# S1400
penicillin-streptomycin solution	Nacalai Tesque	Cat# 26253-84
amphotericin B	Sigma-Aldrich	Cat# A2942-20ML
Opti-MEM (1×)	Gibco	Cat# 31985-062
HBSS(+)	FUJIFILM Wako Pure Chemical	Cat# 084-08965
HEPES buffer	FUJIFILM Wako Pure Chemical	Cat# 345-06681
D-glucose (45 w/v%)	FUJIFILM Wako Pure Chemical	Cat# 079-05511
copper(II) chloride dihydrate	FUJIFILM Wako Pure Chemical	Cat# 038-04122
dimethyl sulfoxide (DMSO)	Tokyo Chemical Industry (TCI)	Cat# D0798
FuGENE 4K Transfection Reagent	Promega Corporation	Cat# E5911
GloSensor cAMP assay / reagent	Promega Corporation	Cat# E1291
Human Genomic DNA: Human Mixed	Promega Corporation	Cat# G3041

Hana3A cells	Literature	N/A
pCI Mammalian Expression Vector	Promega Corporation	Cat# E1731
pHEK293 Ultra Expression Vector	Takara Bio	Cat# 3390
RTP1s vector	Literature	N/A
poly-D-lysine-coated white 96-well plate	Greiner Bio-One	Cat# 655945
Tenax TA tube	Gerstel	Cat# 013741-010-KK
DB-WAX column	Agilent Technologies	J&W 122-7062

Hana3A cells were used according to previously reported heterologous olfactory receptor assay systems [6, 22]. RTP1s was used as an accessory factor for olfactory receptor trafficking and functional expression [21, 23].

Table S3. Previously reported olfactory receptor responses and EC_{50} values relevant to Mix design

Compound	OR10G7	OR5K1	OR2J2
MetEug	Rep.; 1 µM [19], 10.47 µM [20]	Rep.; 10 nM [19], 8.71µM [20], 54.95 nM [44]	Rep.; 14.79 µM [20]
Eug	Rep.; 0.21 µM [20], 10.23 nM [44]	N.C.	Rep.; 15.49 µM [20]
DP	—	Rep.; 2.2 mM [34]	—
Dec	—	—	N.C.

Rep., reported; N.C., not confirmed in the cited literature. MetEug: methyl eugenol; Eug: eugenol; DP: 2,5-dimethylpyrazine; Dec: (*E*,*E*)-2,4-decadienal.

Table S4. EC_{50} values estimated from concentration–response curves in this study

Compound	OR10G7	OR5K1	OR2J2
MetEug	90.3 μM (R^2 = 0.54)	57.8 μM (R^2 = 0.95)	51.5 μM (R^2 = 0.97)
Eug	1.35 μM (R^2 = 0.90)	N.D.	333 μM (R^2 = 0.88)
DP	—	1.47 mM, extrapolated	—
Dec	—	—	48.5 μM (R^2 = 0.92)

EC_{50} values were estimated by nonlinear regression using GraphPad Prism. N.D., not determined because reliable fitting was not obtained. —, not tested or not applicable. Extrapolated values were influenced by high-concentration responses.

Table S5. Previously reported odor descriptors and odor thresholds related to Mix design

Compound	Odor descriptors	OW (mg/L)
MetEug	spicy, clove-like [16]; hay-like, dried-grass [17]; woody, spicy [18]	0.068 [37]
Eug	spicy, clove-like [16]; spicy, cloves, sweet [17]	0.006–0.011 [37]
DP	cocoa, roasted nut, roast beef, medicine [36]	1.7–1.8 [38]
Dec	fatty, waxy, raw, green [36]	0.00007 [37]

OW, odor threshold in water. MetEug: methyl eugenol; Eug: eugenol; DP: 2,5-dimethylpyrazine; Dec: (*E*,*E*)-2,4-decadienal.

Table S6. Composition of the Mix stock solution used for sensory evaluation and olfactory receptor assays

Component in Mix	Concentration in sensory stock (% w/v, ethanol)	Final in-well concentration in receptor assay
Eug	0.150	0.914 mM
DP	0.090	0.833 mM
Dec	5.0×10^{-7}	3.29 μM

The sensory stock concentration indicates the concentration in the ethanol solution used to prepare the sensory evaluation samples. The values for the receptor assay indicate the final in-well stimulus concentrations after the DMSO stock solution was diluted with assay buffer and added to the wells. Eug: eugenol; DP: 2,5-dimethylpyrazine; Dec: (*E*,*E*)-2,4-decadienal.

Table S7. Mean VAS values in the initial main test and presentation concentrations before and after one-point correction

Sample	Mean VAS in Initial Main Test	Initial Main-Test Concentration	Final Presentation Concentration
MetEug	37.34	1	1
Mix	22.77	0.42	0.7
Eug	35.04	0.079	0.084
MetIsoEug	35.32	0.93	0.98
IsoEug	57.84	0.065	0.042
DP	67.74	1.93	1.06
Dec	40.34	0.0000073	0.0000067

Mean VAS values indicate the values obtained in the initial main test before one-point correction. Final presentation concentrations indicate the conditions used for the final VAS and subsequent sensory evaluations after one-point correction. Each value represents the ethanol stock concentration of each sample, except for Mix, which is shown as a dilution factor relative to the defined Mix stock solution. MetEug: methyl eugenol; Eug: eugenol; MetIsoEug: methyl isoeugenol; IsoEug: isoeugenol; DP: 2,5-dimethylpyrazine; Dec: (*E*,*E*)-2,4-decadienal.

Table S8. CATA attribute-wise Cochran's Q test results

Attribute	Q statistic	p-value	Significant
aldehydic	14.0339	0.0293	Yes
clove	32.5200	<0.0001	Yes
sweet	32.6667	<0.0001	Yes
medicinal	19.1087	0.0040	Yes
herbal	12.1176	0.0594	No
resin	8.9600	0.1758	No
phenolic	8.3294	0.2149	No
earthy	28.8649	<0.0001	Yes
vanilla	33.9273	<0.0001	Yes
oily	48.7059	<0.0001	Yes
roasted	61.1538	<0.0001	Yes
fruity	4.3256	0.6327	No
tropical	5.1429	0.5256	No
berry	6.8571	0.3343	No
apple	17.6364	0.0072	Yes
grape	9.8400	0.1316	No
banana	6.3750	0.3825	No
citrus	7.3636	0.2885	No
orange	NC	—	—
lemon	NC	—	—
floral	5.5385	0.4768	No
rose	10.3333	0.1113	No
jasmine	5.0000	0.5438	No
green	3.5000	0.7440	No

leafy	4.5000	0.6093	No
grassy	7.0345	0.3177	No
fresh	6.0000	0.4232	No
minty	11.0000	0.0884	No
woody	7.3333	0.2911	No
pine wood	9.0769	0.1693	No
cedar	10.1538	0.1183	No
amber	3.7091	0.7160	No
spicy	29.1429	<0.0001	Yes
cinnamon	4.2353	0.6449	No
honey	15.5000	0.0167	Yes
caramel	11.7500	0.0678	No
milk	10.3636	0.1102	No
creamy	10.8462	0.0932	No
buttery	16.1538	0.0130	Yes
cocoa	49.5918	<0.0001	Yes
nutty	54.8136	<0.0001	Yes
coffee	46.0000	<0.0001	Yes
smoky	6.6176	0.3577	No
burnt	18.8571	0.0044	Yes
winey	14.8421	0.0215	Yes
waxy	8.8125	0.1844	No
powdery	2.6897	0.8467	No
dry	7.0909	0.3125	No
meaty	12.6000	0.0498	Yes
animalic	4.0000	0.6767	No

painty	11.8824	0.0646	No
metallic	12.0000	0.0620	No
sulfur	6.0000	0.4232	No
onion	6.0000	0.4232	No

NC, not calculated; —, not applicable.

Table S9. Selection counts for representative CATA attributes

Attribute	**MetEug (n/27)**	**Mix (n/27)**	**Eug (n/27)**	**MetIsoEug (n/27)**	**IsoEug (n/27)**	**Dec (n/27)**	**DP (n/27)**
aldehydic	3	0	6	1	2	7	4
clove	7	15	12	4	15	3	6
sweet	0	9	4	2	10	0	3
medicinal	9	9	8	11	4	8	3
herbal	4	5	1	6	1	1	1
resin	6	9	2	5	6	4	3
phenolic	7	10	7	4	8	3	6
earthy	9	1	2	12	5	2	7
vanilla	0	2	0	1	11	2	4
oily	3	0	2	1	2	15	2
roasted	2	1	0	0	1	1	14

Selection counts for representative CATA attributes in the CATA evaluation among 27 panelists. Only the attributes discussed in the main text in relation to the odor shift of Mix are shown. Values represent the numbers of panelists who selected each attribute for each sample.

Table S10. Matrix of Holm-adjusted *p-values* for all 10 pairwise comparisons among the five samples in Napping

	MetEug	Eug	Mix	IsoEug	MetIsoEug
MetEug	—	0.1188	1.0000	0.6134	0.6377
Eug	0.1188	—	0.2460	0.0930	0.1188
Mix	1.0000	0.2460	—	1.0000	0.6134
IsoEug	0.6134	0.0930	1.0000	—	0.1188
MetIsoEug	0.6377	0.1188	0.6134	0.1188	—

Values are Holm-adjusted *p-values* from paired 2D permutation tests. —, self-comparison. MetEug: methyl eugenol; Eug: eugenol; IsoEug: isoeugenol; MetIsoEug: methyl isoeugenol.

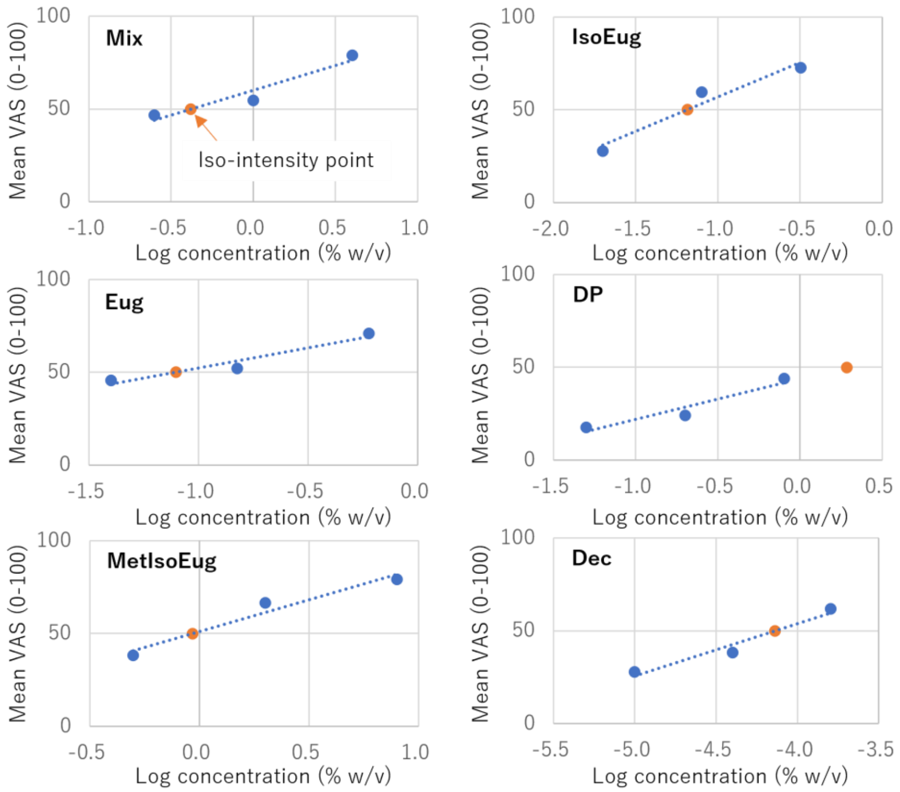

Figure S1. Concentration–response relationships of each sample in the preliminary VAS. After MetEug was first presented as the reference (= 50), the relative intensity of each sample was evaluated at three concentration or dilution levels (n = 8 panelists). The mean VAS value for each sample was plotted against log concentration, and an approximate iso-intensity point was estimated from this relationship to set the concentration for the initial main test. MetEug: methyl eugenol; Eug: eugenol; MetIsoEug: methyl isoeugenol; IsoEug: isoeugenol; DP: 2,5-dimethylpyrazine; Dec: (*E*,*E*)-2,4-decadienal.

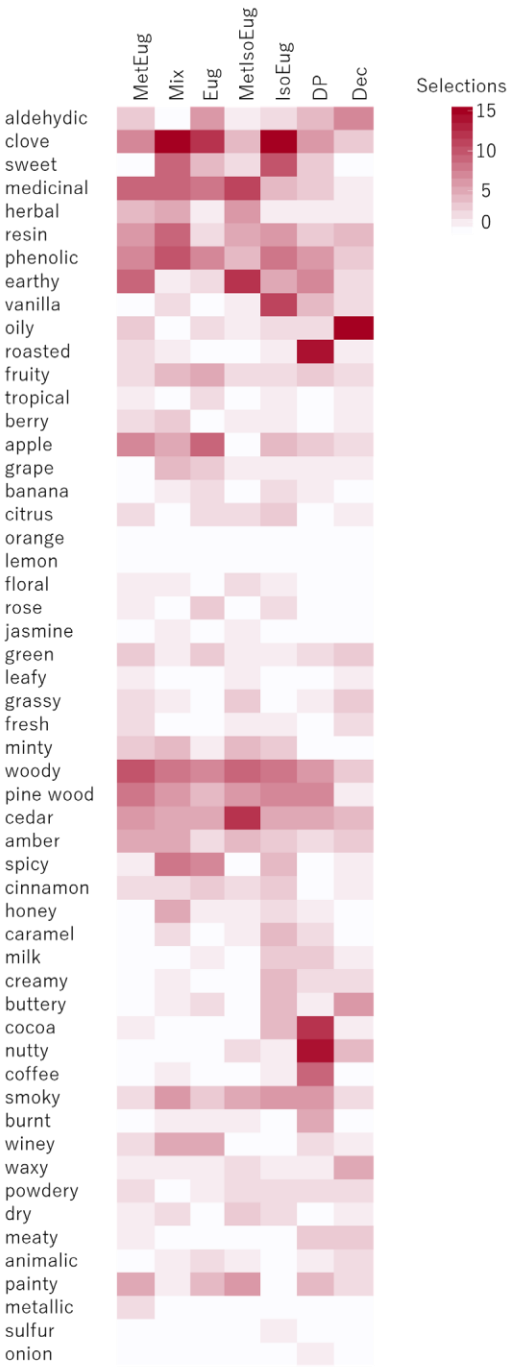

Figure S2. CATA selection-frequency heatmap for all 54 attributes. Rows indicate the 54 CATA attribute terms, and columns indicate the seven evaluated samples (MetEug, Mix, Eug, MetIsoEug, IsoEug, DP, and Dec). The color of each cell indicates the selection frequency (%) for the corresponding attribute term, with darker colors representing higher selection frequencies (n = 27 panelists). The attribute terms were arranged with the major terms discussed in the main text placed first, followed by grouping according to similar odor characters. MetEug: methyl eugenol; Eug: eugenol; MetIsoEug: methyl isoeugenol; IsoEug: isoeugenol; DP: 2,5-dimethylpyrazine; Dec: (*E*,*E*)-2,4-decadienal.

Supplementary Note S1. Napping GPA analysis environment and analysis settings

Napping GPA analysis was performed in a Python 3.11.15 environment. The main packages used were NumPy 2.4.6, Pandas 3.0.3, and Matplotlib 3.10.9. In the main analysis, the coordinates of MetEug1 and MetEug2 were averaged within each panelist and used as MetEug. Seven samples (MetEug, MetIsoEug, Eug, IsoEug, Dec, DP, and Mix) were included in the analysis. In GPA, translation, rotation, and isotropic scaling were performed, whereas reflection was not allowed (MAX_ITER = 500, TOL = 1e-12).
